# Drug Utilization and Inappropriate Prescribing in Centenarians

**DOI:** 10.1111/jgs.14106

**Published:** 2016-04-30

**Authors:** Nisha C. Hazra, Alex Dregan, Stephen Jackson, Martin C. Gulliford

**Affiliations:** ^1^ Department of Primary Care and Public Health SciencesKing's College LondonLondonUK; ^2^ Biomedical Research CentreNational Institute for Health ResearchGuy's and St Thomas’ National Health Service Foundation TrustLondonUK; ^3^ Department of Clinical GerontologyKing's College HospitalLondonUK

**Keywords:** centenarians, epidemiology, inappropriate prescribing, aging, primary care

## Abstract

**Objectives:**

To use primary care electronic health records (EHRs) to evaluate prescriptions and inappropriate prescribing in men and women at age 100.

**Design:**

Population‐based cohort study.

**Setting:**

Primary care database in the United Kingdom, 1990 to 2013.

**Participants:**

Individuals reaching the age of 100 between 1990 and 2013 (N = 11,084; n = 8,982 women, n = 2,102 men).

**Measurements:**

Main drug classes prescribed and potentially inappropriate prescribing according to the 2012 American Geriatrics Society Beers Criteria.

**Results:**

At the age of 100, 73% of individuals (79% of women, 54% of men) had received one or more prescription drugs, with a median of 7 (interquartile range 0–12) prescription items. The most frequently prescribed drug classes were cardiovascular (53%), central nervous system (CNS) (53%), and gastrointestinal (47%). Overall, 32% of participants (28% of men, 32% of women) who received drug prescriptions may have received one or more potentially inappropriate prescriptions, with temazepam and amitriptyline being the most frequent. CNS prescriptions were potentially inappropriate in 23% of individuals, and anticholinergic prescriptions were potentially inappropriate in 18% of individuals.

**Conclusion:**

The majority of centenarians are prescribed one or more drug therapies, and the prescription may be inappropriate for up to one‐third of these individuals. Research using EHRs offers opportunities to understand prescribing trends and improve pharmacological care of the oldest adults.

Remarkable reductions in mortality are associated with rapid increases in life expectancy in many developed countries. Recent estimates in the United Kingdom (UK) indicate a quintupling of the number of centenarians over the past 3 decades, to 13,780 in 2013.^1^ This trend is expected to continue, with approximately 68 #bib000 centenarians in the United Kingdom^2^ and 1 million centenarians worldwide by 2030.^3^, ^4^ Greater longevity is likely to be associated with higher rates of drug prescribing and greater expenditures on health services.^5^ Elderly people consume 45% to 55% of all drugs in the United Kingdom,^6^, ^7^ and 38% of all drugs in the United States,^8^ but few studies in the United Kingdom have described drug utilization and none have described potentially inappropriate prescribing (PIP) in the oldest adults.

Drug prescribing and managing medication use in older adults is complex and challenging because multiple morbidity, frailty, and cognitive impairment often accompany old age. Drug prescribing in very old adults is challenging to clinicians because very old adults often require multiple drug therapy but are also at great risk of drug‐related adverse effects. Evidence regarding drug prescribing in centenarians is rare and, when available, rather inconsistent.^9^, ^10^, ^11^, ^12^ Existing studies include self‐reported data and tend to focus on younger old people,^7^, ^9^, ^10^, ^12^ often excluding centenarians.^9^, ^13^, ^14^

Electronic health records (EHRs) are a valuable resource to explore the epidemiology of drug prescribing in population‐based samples.^12^ Electronic data can help overcome biases in self‐reported prescribing patterns and medical histories, a common limitation of previous studies.^7^, ^15^, ^16^, ^17^ The central role of general practice within the U.K. National Health Service allows for long‐term prescribing to be managed in primary care. This study aimed to use primary care EHR data from the U.K. Clinical Practice Research Datalink (CPRD), one of the world's largest primary care databases, to describe drug use and to estimate the frequency of PIP in a large cohort of centenarians.

## Methods

### Setting and Study Design

The study included a nationally representative sample of individuals in primary care from the CPRD who reached the age of 100 between 1990 and 2013, as described previously.^18^ Practices can contribute data only when it is deemed of sufficient quality for research, according to a set of criteria that the CPRD group has developed.^19^ This study, which received scientific and ethical approval from the Independent Scientific Advisory Committee for CPRD studies (ISAC Protocol 13_151), is based on fully anonymized data, and research ethics committee approval was not required.

### Participants

A population‐based cohort of centenarians was drawn from the CPRD between January 1 #bib1990, and September 30, 2013, as previously described.^18^ Eligible participants were registered at CPRD general practices during the year in which they turned 100.

### Measures

Drug utilization was first evaluated using the main chapters from the British National Formulary (BNF) as categories. The BNF is published every 6 months and provides healthcare professionals with up‐to‐date information about medicines.^20^ Each participant's record was evaluated for drug prescriptions in the following drug categories: gastrointestinal; cardiovascular; respiratory; central nervous system (CNS); antimicrobial; endocrine; gynecological and urinary tract; neoplasms and immunosuppression; nutrition and blood; musculoskeletal; eye; ear, nose, and oropharynx; skin; immunological; and anesthetic. Centenarians were considered to have been prescribed a drug from a particular category if at least one relevant drug was prescribed during their 100th year. Drugs relevant to more than one category were counted in each. For example, prednisolone is included in the endocrine and gastrointestinal categories. Descriptive statistics were used to estimate the total number of prescriptions before reaching the age of 100, from the 95th year to the 100th year of life.

The frequency of PIP among centenarians was evaluated using the updated 2012 American Geriatrics Society (AGS) Beers Criteria.^21^ An expert group developed the criteria in 1991,^22^ with the latest revision by the AGS in 2012,^21^ to provide explicit criteria for PIP in elderly adults. The criteria include three primary groups: a list of potentially inappropriate medication (PIMs) to avoid independent of disease or condition; a list of medications to be avoided in older persons with specific diseases or conditions; and a list of PIMs to be used with caution in older adults.^21^ This tool has been widely used to evaluate PIP of drugs to elderly individuals in a variety of settings.^15^, ^16^, ^17^, ^23^, ^24^, ^25^

In the present study, we estimated the proportion of centenarians receiving one or more prescriptions from each overall Beers category, independent of disease or condition, including—anticholinergics, anti‐thrombotics, anti‐infectives, cardiovascular, CNS, endocrine, gastrointestinal, and analgesics. The proportion prescribed at least one specific PIM within each category was also estimated. The standard criteria were used, rather than the disease‐specific criteria, because of the large population‐based sample in the study. The three drugs that were most frequently prescribed inappropriately were evaluated, and descriptive statistics were used to determine the median number of PIMs in each category. Desiccated thyroid is not listed in the BNF, so it was excluded from the analysis. Glyburide is known as glibenclamide in the United Kingdom. The prescribing of nonsteroidal anti‐inflammatory drugs (NSAIDs) in this study was considered inappropriate only if, as stated in the 2012 AGS Beers Criteria, they were not taken with a gastroprotective agent. All topical non‐cyclooxygenase‐selective NSAIDs were not considered as inappropriate and only oral formulations were included, as stated by the 2012 AGS Beers Criteria. Stata version 13.0 was used to conduct all analysis (Stata Corp., College Station, TX).

## Results

A cohort of 11,047 centenarians (8,982 women #bib2 #bib102 men), who reached the age of 100 between 1990 and 2013, was selected for analysis. Eighty‐four percent were born between 1900 and 1913 and the remaining 16% between 1890 and 1899. The median annual number of prescriptions was 7 (interquartile range 0–12) during the 100th year.

**Figure 1:**
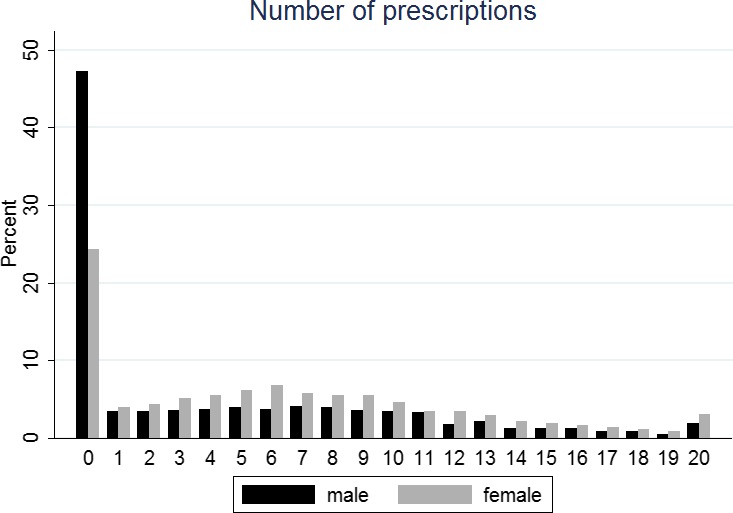
Frequency of prescriptions during 100th year according to sex.

Table 1 shows the most frequently prescribed drugs at the age of 100. Drug utilization for each drug class was higher in women than in men, except for urinary tract drugs. The most frequently prescribed drugs overall were those affecting the CNS (53%), including hypnotics, anxiolytics, antidepressants, analgesics, drugs for nausea and vertigo, antiepileptics, and antidementia, as well as cardiovascular (53%) and gastrointestinal (47%) drugs. The distribution of prescriptions issued to men and women centenarians is shown in Figure 1. More than twice as many men as women did not receive any prescriptions in their 100th year, and the proportion of women was higher than men for each increasing category of multiple prescriptions.

**Table 1:** Frequency of Different Categories of Prescriptions According to Sex

Drug Class from British National Formulary	Female, n = 8,982	Male, n = 2,102	All, N = 11,084
	n (%)^a^		
Any	6,904 (79)	1,136 (54)	8,040 (73)
Cardiovascular	5,044 (56)	835 (40)	5,879 (53)
Central nervous system^b^	5,182 (58)	741 (35)	5,923 (53)
Gastrointestinal	4,488 (50)	752 (36)	5,240 (47)
Skin	3,524 (39)	537 (26)	4,061 (37)
Nutrition and blood	3,485 (39)	479 (23)	3,964 (36)
Antimicrobial	4,028 (45)	631 (30)	4,659 (42)
Eye	2,038 (23)	316 (15)	2,354 (21)
Musculoskeletal and joint diseases	1,963 (22)	307 (15)	2,270 (20)
Endocrine	1,818 (20)	283 (13)	2,101 (19)
Respiratory	1,545 (17)	277 (13)	1,822 (16)
Anesthesia	1,131 (13)	198 (9)	1,329 (12)
Ear, nose, oropharynx	828 (9)	171 (8)	999 (9)
Immunological products and vaccines	786 (9)	119 (6)	905 (8)
Gynecological and urinary tract	692 (8)	206 (10)	898 (8)
Neoplasms and immunosuppression	497 (6)	92 (4)	589 (5)

a Frequency of individuals with at least one prescription during the year they turned 100.

b Hypnotics, anxiolytics, antidepressants, analgesics, drugs for nausea and vertigo, antiepileptics, drugs for parkinsonism and dementia.

The frequency of PIP according to the 2012 AGS Beers Criteria is presented in Table 2. Overall, 32% of centenarians were prescribed a PIM, and the three most frequently prescribed PIMs were temazepam, amitriptyline, and nitrofurantoin. The drug class with the highest proportion of PIP was CNS medications (23%). Approximately one‐fifth of centenarians (19% women, 13% men) received anticholinergic drugs, with PIMs including chlorphenamine, hydroxyzine, and promethazine hydrochloride. Despite the high levels of prescribing for gastrointestinal, cardiovascular, and analgesic drugs, low levels of inappropriate prescribing (5%) were observed within these classes.

**Table 2:** Frequency of Potentially Inappropriate Medications Using 2012 American Geriatrics Society (AGS) Beers Criteria

Drug Category	Female, n = 8,982				Male, n = 2,102				All, N = 11,084			
	Total Drug Class, n^a^	PIM, n (%)^b^	Number of PIMs, Median (IQR)	Top 3 PIMs	Total Drug Class, n^a^	PIM, n (%)^b^	Number of PIMs, Median (IQR)	Top 3 PIMs	Total Drug Class, n^a^	PIM, n (%)^b^	Number of PIMs, Median (IQR)	Top 3 PIMs
Any	6,799	2,208 (32)	5 (2–12)	Temazepam Amitriptyline Nitrofurantoin	1,108	309 (28)	4 (1–10)	Temazepam Dipyridamole Amitriptyline	7,907	2,517 (32)	5 (2–12)	Temazepam Amitriptyline Nitrofurantoin
Central nervous system	5,182	1,240 (24)	7 (2–13)	Temazepam Amitriptyline Diazepam	741	147 (20)	5 (2–12)	Temazepam Amitriptyline Diazepam	5,923	1,387 (23)	6 (2–12)	Temazepam Amitriptyline Diazepam
Anticholinergic	1,279	237 (19)	2 (1–8)	Chlorphenamine Hydroxyzine Promethazine hydrochloride	219	28 (13)	2 (1–6)	Chlorphenamine Promezathine hydrochloride Hydroxyzine	1,498	265 (18)	2 (1–7)	Chlorphenamine Hydroxyzine Promethazine hydrochloride
Anti‐infective	4,027	353 (9)	1 (1–2)	Nitrofurantoin	631	38 (6)	1 (1–2)	Nitrofurantoin	4,658	391 (8)	1 (1–2)	Nitrofurantoin
Analgesic	5,348	258 (5)	2.5 (1–7)	Ibuprofen Diclofenac Meloxicam	844	31 (4)	1 (1–2)	Ibuprofen Diclofenac Meloxicam	6,192	289 (5)	2 (1–6)	Ibuprofen Diclofenac Meloxicam
Gastrointestinal	4,653	238 (5)	1 (1–4)	Liquid paraffin (mineral oil) Metoclopramide	773	31 (4)	1 (1–2)	Metoclopramide Liquid paraffin (mineral oil)	5,426	269 (5)	1 (1–3)	Liquid paraffin (mineral oil) Metoclopramide
Cardiovascular	5,044	207 (4)	7 (3–13)	Amiodarone Doxazosin Sotalol	835	50 (6)	6 (3–11)	Amiodarone Doxazosin Spironolactone	5,879	257 (4)	7 (3–13)	Amiodarone Doxazosin Spironolactone
Antithrombotic	2,578	87 (3)	6 (2–12)	Dipyridamole	460	26 (6)	6 (2–12)	Dipyridamole	3,038	113 (4)	6 (2–12)	Dipyridamole
Endocrine	1,888	15 (1)	11 (3–13)	Glyburide Conjugated estrogens Megestrol	311	1 (0.3)	6 (6–6)	Glyburide	2,199	16 (0.1)	9 (4–13)	Glyburide Conjugated estrogens Megestrol

a Number of individuals from total sample with at least one prescription during the year of turning 100 years old in each overall Beers category.

b PIMs = potentially inappropriate medications within each Beers category as defined by the 2012 AGS Beers Criteria (row percentages).

## Discussion

To the knowledge of the authors, this is the first population‐based study describing medication use and PIP in centenarians. Only a minority of centenarians did not receive prescription medicines, with a higher proportion of men not receiving any prescriptions, consistent with their superior health status at age 100.^18^ Almost 80% of women and 54% of men were prescribed at least one drug during their 100th year. Sex differences in overall prescribing between centenarians have not been reported previously. This disparity could be because of sex differences in health‐seeking behavior, for example, not seeking a physician's advice, or nonadherence, as well as resulting from differences in health status. Up to one‐third of centenarians were prescribed a PIM. The highest frequencies of PIP were attributed to the use of benzodiazepines (temazepam, diazepam), amitriptyline, and nitrofurantoin. The 2012 AGS Beers Criteria recommendation to avoid all of these drugs is “strong,” and the reported quality of evidence is “high” for avoiding temazepam, diazepam, and amitriptyline and “moderate” for avoiding nitrofurantoin.^21^ The 2012 AGS Beers Criteria recommend avoiding nitrofurantoin in individuals with creatinine clearance less than 60 mL/min because of concerns about lack of efficacy from inadequate drug concentrations in the urine. In view of the advanced age of the cohort, it is likely that most will have poor renal function and should therefore be using safer alternatives such as ciprofloxacin or trimethoprim.^26^

### Comparison with Existing Literature

Existing studies on drug utilization in elderly adults have often focused on younger cohorts of old people^7^, ^9^, ^10^, ^12^ or do not include centenarians.^9^, ^13^, ^14^ There is scarce evidence about PIP in extreme old age. These studies focusing on younger elderly adults tend to rely on self‐reported questionnaires and interviews,^7^, ^15^, ^16^, ^17^, ^24^, ^25^, ^26^, ^27^ resulting in a high risk of responder bias.

There have been no previous studies reporting PIP in a large group of centenarians. Reports of prevalence of PIP in individuals aged 65 and older are inconsistent, and few studies have used the updated 2012 criteria. One study^27^ using the 2012 criteria reported a higher prevalence of PIP (44%) in Spanish individuals aged 65 and older than the present findings (32%) in centenarians. The most frequently prescribed PIMs were benzodiazepines, similar to the present data. Another study in New Zealand^24^ also reported a higher PIM prevalence (42.7%) for community‐dwelling individuals aged 75 and older than for those aged 100 and older in CPRD, whereas another^15^ reported a prevalence of 17%. All three studies^15^, ^24^, ^27^ used self‐reported data. Several studies reported pain medications as the most commonly prescribed PIMs,^15^, ^17^, ^23^, ^24^ including two studies using the Screening Tool of Older People's potentially inappropriate Prescriptions/Screening Tool to Alert doctors to the Right Treatment (STOPP/START) criteria.^28^, ^29^ This is inconsistent with the present findings in centenarians, reporting CNS medications and anticholinergics as the most commonly prescribed PIMs. It is not stated in all these previous studies whether concurrent use of gastroprotective agents was considered alongside NSAIDs.

### Strengths and Limitations

This study had the strengths of a large sample drawn from a representative population of U.K. general practices. In the United Kingdom, approximately 98% of individuals are registered with a family practice, ensuring that the present data are complete and nationally representative. Individuals aged 75 and older have an annual review of medicines,^30^ and those with four or more medicines are reviewed every 6 months, although this review was not introduced until 2002. Using primary care EHRs allowed for the classification of prescribing according to drug category and of specific PIMs, but data were not available for several variables of interest, including whether an individual lived alone or whether they lived in an urban or rural location. EHRs circumvent the problem of recall bias, a limitation of many drug use studies relying on self‐reported questionnaires or interviews to collect data on prescriptions. There is a possibility that some prescriptions will not be filled or consumed, and the data may not capture any over‐the‐counter or secondary care hospital prescriptions, so the present findings may underestimate drug usage of centenarians. Another limitation is that the sample was exposed to a nonuniform drug formulary because the study considers data over a 23‐year span. Medical practice in primary care has evolved over time, and as a result, certain drugs used in 1990 may now be considered inappropriate. Several new drugs have also been introduced that could not be prescribed in 1990.

There are limitations of the 2012 AGS Beers Criteria, given their universal application without careful consideration of an individual's response to each drug.^15^, ^25^ There may also be some drugs used in the United Kingdom that were not captured. In 2003, the STOPP/START criteria were developed to identify potential errors in prescribing and prescribing omission in older people according to physiological system.^24^, ^28^ These criteria were created as a Europe‐focused tool but are not as widely used as the 2012 AGS Beers Criteria to evaluate the epidemiology of PIP because of their specificity. Although a more‐individualized tool may be favored in clinical practice, this approach is less feasible for epidemiological investigation of population‐based samples. The 2012 AGS Beers Criteria were specifically designed to use pharmacy records with minimal additional clinical information so that they could be applied to chart reviews or computerized data sets.^21^

According to the 2012 AGS Beers Criteria, the most commonly prescribed PIMs in the present cohort were benzodiazepines, tertiary tricyclic antidepressants, nitrofurantoin, and ibuprofen. The frequencies of PIP found in this study should be interpreted cautiously because each person's risk:benefit ratio for a drug will depend on his or her physiological and clinical status. Only an individual evaluation of each person will confirm the validity of these interpretations. Nevertheless, population‐based studies provide useful epidemiological data on relative frequencies of PIP in large cohorts and help identify the inappropriate medications that are most frequently prescribed. This study also identifies specific drugs that may be given more attention in further research on the determinants of PIP in elderly adults.

### Conclusion and Implications for Clinical Practice

Polypharmacy and multimorbidity in elderly adults present health professionals with a significant clinical responsibility. The limited empirically based evidence to guide drug prescription in very old adults means that physicians must base their prescribing decisions on clinical knowledge and prior experience with similar conditions, likely from younger cases. There is an urgent need for studies to explore the efficacy, safety, and harms associated with drug prescribing for different chronic conditions in very old adults. There is also the need to model the adverse clinical and economic consequences of inappropriate therapeutic decision‐making in this group.

This is the first study to use primary care EHR data to describe prescribing trends in UK centenarians, as well as the extent of PIP according to drug class. It provides proof of concept for using a large EHR database to evaluate appropriateness of prescribing and a basis for reevaluating indicators of appropriate prescribing as applied to this age group. This also offers valuable data for the modeling of future healthcare needs and costs of the oldest adults in the United Kingdom.
